# Perceptions of poverty and associated factors among first- and second-year nursing students: a cross-sectional study

**DOI:** 10.3389/fpubh.2026.1941236

**Published:** 2026-09-23

**Authors:** Neşe Ataman Bor, Soner Berşe, Revşen Özer

**Affiliations:** 1 Department of Nursing, Faculty of Health Sciences, Hakkari University, Hakkari, Türkiye; 2 Department of Nursing, Faculty of Health Sciences, Gaziantep University, Gaziantep, Türkiye; 3 Institute of Social Sciences, Hakkari University, Hakkari, Türkiye

**Keywords:** causal attribution, maternal education, nursing students, poverty perception, social determinants of health, social justice

## Abstract

**Background:**

Poverty is a key social determinant of health. This study examined perceptions of poverty among first- and second-year nursing students and the characteristics associated with them.

**Methods:**

A cross-sectional survey was conducted at a single university in southeastern Türkiye between June and July 2025. All 675 first- and second-year students were invited to complete an anonymous online questionnaire; 523 questionnaires were returned, 519 (76.9%) came from students in an eligible year, and 512 (75.9%) met all eligibility criteria and were analysed. Measures were a personal information form, including a single-response item on the perceived cause of poverty, and the Perception of Poverty Scale for University Students (PPS). Analyses were exploratory: descriptive statistics, t-tests and one-way ANOVA with effect sizes and post-hoc tests, and hierarchical multiple linear regression with heteroscedasticity-consistent (HC3) standard errors.

**Results:**

Poverty was most often attributed to “injustice” (43.0%) and “lack of opportunity” (27.5%). Students selecting “injustice” had higher PPS scores than those selecting “lack of opportunity” (*p* = 0.001, g = 0.41) or “sometimes people are to blame” (*p* = 0.023, g = 0.46). Scores differed by mother’s education (*F* (5, 506) = 3.27, *p* = 0.006, η^2^p = 0.031) but not by father’s education, family income, year of study or employment status. In the final regression model (R^2^ = 0.075), mother’s education (B = −1.15, 95% CI − 1.77 to −0.53) and attribution to injustice (B = 2.88, 95% CI 1.62 to 4.15) were associated with PPS scores; the female–male difference was small and imprecisely estimated (B = 1.29, 95% CI − 0.11 to 2.69), and household income adequacy, year of study and father’s education showed no evidence of association.

**Conclusion:**

In this single-university sample, maternal education and attribution of poverty to injustice were associated with PPS scores, whereas no evidence of an association with self-reported household income adequacy was observed. The analyses were exploratory and the model accounted for a small proportion of the variance; the cross-sectional design does not support causal inference or conclusions about the effectiveness of educational approaches. Poverty-related beliefs merit evaluation in educational research using measures of knowledge, stigma and practice.

## 1 Introduction

Poverty is a multidimensional condition involving financial hardship and deprivations in health, education and living standards (1, 2). Unequal access to resources and opportunities, including adequate housing, education and social protection, contributes to avoidable health inequalities (2). In a longitudinal analysis of US adults, adverse social determinants, including low income and limited education, were associated with shorter life expectancy (3).

For nursing, addressing these inequalities requires attention to patients’ social needs alongside their clinical needs. The National Academies’ report on the future of nursing emphasises social-needs assessment, advocacy and the integration of health equity into nursing education (4). In a qualitative study, nurses described adapting care and patient education to patients’ circumstances and advocating better coordination of services (5). This professional context provides a rationale for examining students’ assumptions about individual responsibility and social constraints within teaching on communication, care planning and advocacy.

Previous research has examined related but distinct beliefs and attitudes. A Canadian study found that greater educational exposure to poverty and more favourable attitudes towards people living in poverty were associated with stronger endorsement of structural explanations of the poverty–health relationship (6). In Türkiye, nursing students endorsed fatalistic explanations of poverty more strongly than social-work students (7). A UK–US comparison examined attitudes towards poverty and social justice, reporting more favourable attitudes in the US sample (8). Among adults in Istanbul, Ankara and Izmir, higher education was associated with structural rather than fatalistic explanations of poverty (9). These findings support considering educational and socioeconomic characteristics, while differences in populations and measures limit direct comparisons.

Poverty simulations have also been used to address students’ beliefs. Comparative and qualitative studies report changes in selected self-reported attitudes and reflection on empathy and social justice (10, 11). However, findings are outcome-dependent: one study found improvement in social-justice attitudes only in the non-simulation comparison group (12). These studies provide an educational context for examining existing beliefs, but their varied designs and reliance on self-report limit conclusions about changes in clinical practice.

In this study, ‘perceptions of poverty’ refers to the beliefs operationalised by the Perception of Poverty Scale for University Students (PPS), including relative and social definitions of poverty and economic, political and migration-related explanations (13). This operational definition distinguishes the beliefs examined here from factual knowledge about poverty and evaluative attitudes towards people living in poverty. Higher PPS scores indicate stronger endorsement of its statements, not necessarily more accurate knowledge or less stigma. It remains unclear how PPS-defined perceptions relate to students’ selected explanation of poverty when family income adequacy and both parents’ education are considered together in early-year nursing students.

The study therefore aimed to describe perceptions of poverty among first- and second-year nursing students at one university in Gaziantep, Türkiye, and to explore the associations of gender, year of study, family income adequacy, maternal education, paternal education and the selected cause of poverty with PPS scores in a joint model. Its contribution is a context-specific, exploratory examination of these relationships; it does not evaluate educational effectiveness, clinical judgement, communication or advocacy outcomes.

## 2 Methods

### 2.1 Design, setting and participants

This cross-sectional descriptive study was conducted at the Department of Nursing, Gaziantep University, Türkiye, between June and July 2025. All students enrolled in the first- and second-year cohorts were invited to participate; these were the cohorts accessible to the research team through class communication channels, and no claim is made about their curricular or clinical exposure relative to senior cohorts. Participation was voluntary and anonymous; the analysed group is therefore a self-selected sample of two purposively chosen cohorts.

The department enrolled 1,088 undergraduate students, of whom 675 were in the two cohorts (year 1, *n* = 385; year 2, *n* = 290). Eligibility criteria were enrolment in year 1 or 2 at the time of data collection, age 18 years or over, and consent to participate.

Students were invited through class WhatsApp groups, Instagram and Facebook student groups, and institutional e-mail. A total of 523 questionnaires were returned. Four were excluded because the respondent reported enrolment in year 3 or 4. Seven were excluded on the age criterion: four respondents were aged under 18, one entered an invalid age, and two entered text that was not an age, so that their eligibility could not be verified. One date-formatted age entry was interpreted as a date of birth consistent with adult eligibility, and the record was retained. Because exact age at participation could not be established, this record was excluded only from the descriptive age summary. The analytic sample was 512 (Figure 1). Because the invitation was posted to group channels, individual receipt cannot be verified and students who declined cannot be distinguished from those who did not respond; 519/675 (76.9%) is therefore reported as the proportion of students in the two cohorts who returned a questionnaire and 512/675 (75.9%) as the proportion analysed, not as response rates. Participation was higher in year 1 (338/385, 87.8%) than in year 2 (181/290, 62.4%; χ^2^(1) = 59.95, *p* < 0.001).

**Figure 1:**
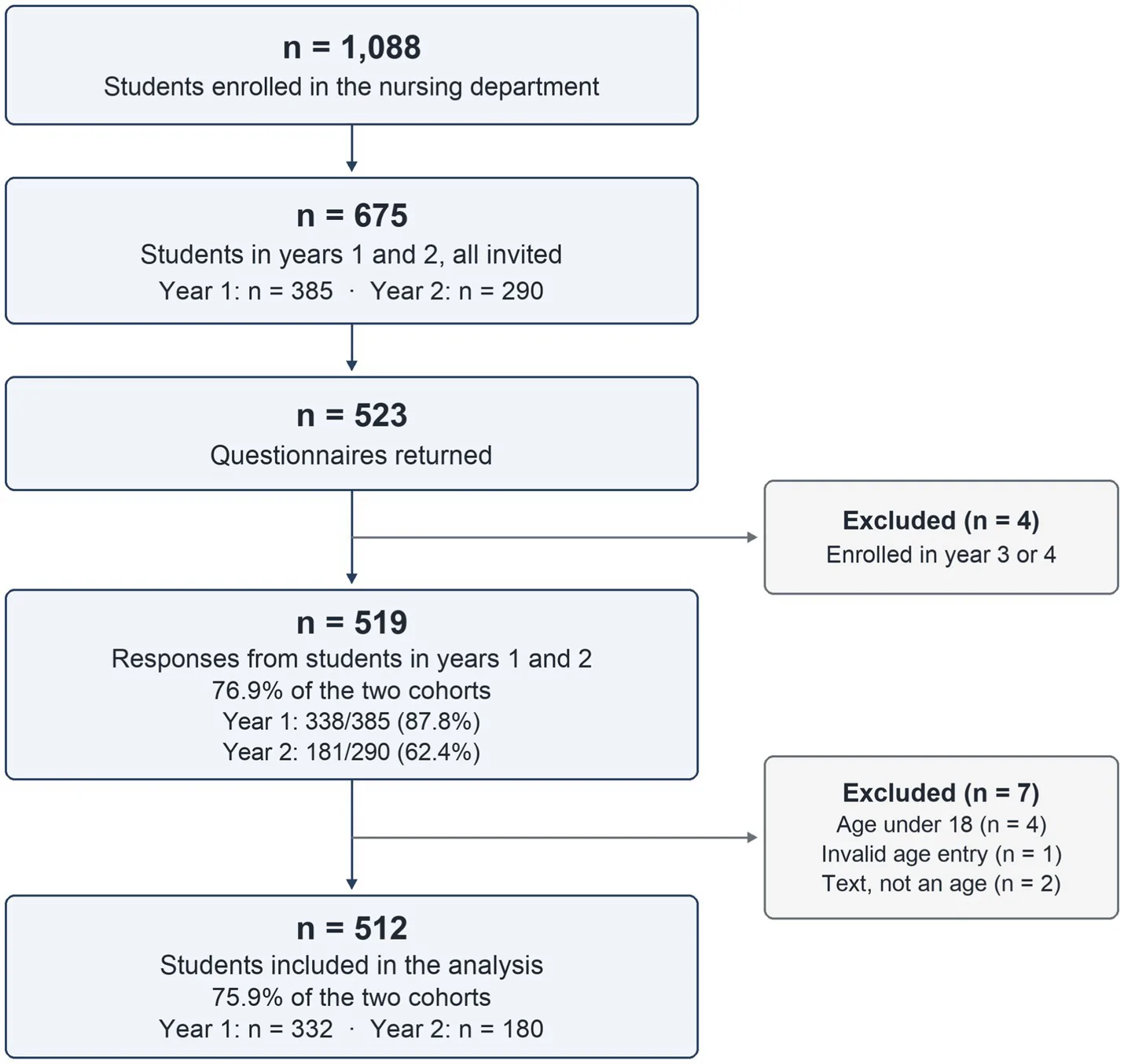
Participant flow. Overall percentages use the 675 students in the two cohorts; year-specific percentages use the respective cohort sizes.

No *a priori* sample-size calculation was performed. All students in the two selected cohorts were invited, and the final analytic sample size was determined by voluntary participation and eligibility screening. A sensitivity analysis for the conventional overall regression F-test, with 512 observations and six predictors, indicated a minimum detectable effect of f^2^ ≈ 0.027 (R^2^ ≈ 0.026) at 80% power and α = 0.05. This calculation was not an *a priori* sample-size justification, and no power estimate based on the observed effect was reported.

### 2.2 Measures

#### 2.2.1 Personal information form

A researcher-prepared form recorded age, gender, employment status, year of study, family income level, mother’s and father’s education level, and parental employment status. It also included a single item on the perceived cause of poverty with five mutually exclusive options — “personal problems/illness,” “injustice,” “lack of opportunity”, “fate” and “sometimes people are to blame”. This item was single-response: each participant selected exactly one option, and the five category frequencies sum to the analytic sample. Percentages are therefore mutually exclusive and total 100%. The same category labels are used consistently wherever they are reported.

#### 2.2.2 Perception of poverty scale for university students

The Perception of Poverty Scale for University Students (PPS), developed by Uru and Dirimeşe (13), was used. The scale was developed and validated in Turkish with a Turkish university-student population, so no cross-cultural adaptation was required. Its two-factor structure — Socio-Economic and Political (items 3, 4, 5, 6, 7, 9, 10) and Work and Employment (items 1, 2, 8, 11) — was established by exploratory and confirmatory factor analysis in the development study, where Cronbach’s α was 0.809. Responses are scored 1–5 with no reverse-scored items, giving ranges of 11–55 for the total score and 7–35 and 4–20 for the Socio-Economic and Political and the Work and Employment subscales. Higher scores indicate greater agreement with the scale’s statements, which define poverty in relative and social terms (for example, working for the minimum wage or being unable to take part in social activities) and attribute it to macro-level economic, political and migration-related causes; scores are not interpreted as indicating knowledge, empathy or professional competence. Written permission was obtained from the developers.

In the present sample, internal consistency was α = 0.908 (95% CI 0.895–0.919) for the total scale, 0.869 for the Socio-Economic and Political subscale and 0.779 for the Work and Employment subscale. Corrected item–total correlations ranged from 0.52 to 0.74, with no item below the conventional 0.30 threshold. A confirmatory factor analysis of the two-factor model in the present sample (maximum likelihood, items treated as continuous) gave χ^2^(43) = 213.3, CFI = 0.94, TLI = 0.92, RMSEA = 0.088 and SRMR = 0.041 (root mean square of the standardised residuals over all unique elements of the covariance matrix), with standardised loadings of 0.57–0.79; the correlation between the two factors was 0.96, and the two subscale scores correlated 0.80. Subscale scores are therefore reported as secondary outcomes and are not interpreted as distinct constructs.

### 2.3 Data collection and ethics

Ethical approval was obtained from the Hakkari University Ethics Committee (Decision No: 2025/109), and institutional permission was obtained from Gaziantep University. Written permission was obtained from the scale developers. Participants were informed about the purpose and scope of the study, that participation was voluntary, and that data would be used solely for scientific purposes; consent was obtained online before the questionnaire was completed. The questionnaire was administered through Google Forms, no identifying information was requested, and completion took approximately 10–15 min. The analysis was restricted to participants meeting the adult eligibility criterion; records that did not meet this criterion, or for which it could not be verified, were excluded (Section 2.1, Figure 1).

### 2.4 Data analysis

Analyses were performed in Python 3.12 using NumPy, pandas, SciPy, statsmodels and pingouin. Confirmatory factor analysis used maximum likelihood with items treated as continuous and a custom SciPy implementation. The PPS total score was treated as the primary outcome and subscale scores as secondary outcomes. No hypotheses were preregistered; all analyses were exploratory, and the regression model was specified after initial analyses had been examined.

Participant characteristics, PPS scores and poverty explanations were summarised using frequencies (%) and means (SD). Group differences were assessed using independent-samples t-tests or one-way ANOVA. Distributional shape was examined using histograms, Q–Q plots, skewness and kurtosis; median-centred Levene tests assessed variance homogeneity. Welch tests were used for unequal variances, and Tukey HSD for homoscedastic pairwise comparisons. Effect sizes were Hedges’ g with 95% confidence intervals (CIs) and partial η^2^ with 90% CIs. The latter were calculated by inversion of the noncentral F distribution from conventional ANOVA (14). Pearson and Spearman correlations examined parental education and PPS scores.

Hierarchical multiple linear regression with six predictors examined adjusted associations with total PPS scores, not causal effects or individual prediction. Blocks comprised cohort characteristics (gender and year of study), family socioeconomic indicators (income adequacy and maternal and paternal education), and the selected explanation of poverty. Gender and socioeconomic variables were informed by research on poverty attributions (9, 15), while year of study distinguished the sampled cohorts. The attribution item was included to examine how students’ selected explanation of poverty related to PPS scores after accounting for cohort characteristics and family socioeconomic indicators. All predictors were retained irrespective of significance. Coding was female versus male, study year as 1 or 2, income below versus equal to/above expenses, parental education as 1–6, and injustice versus other explanations. The final contrast was exploratory, not a validated structural/non-structural classification.

Sensitivity analyses used three-category income and categorical maternal education and poverty attribution (Supplementary Tables S1, S2). An HC3-based Wald test evaluated departure from linear maternal-education coding. Regression diagnostics included residual distributions, variance inflation factors, Cook’s distance and the Breusch–Pagan test. Coefficient inference used heteroscedasticity-consistent HC3 standard errors (16), with 95% CIs and *p*-values based on the t distribution with residual degrees of freedom. Unstandardised coefficients, standardised point estimates, R^2^, adjusted R^2^ and ΔR^2^ were reported; model and block-comparison F tests used conventional ordinary least squares.

Tukey adjustment applied within each set of pairwise comparisons; other analyses and effect-size CIs were not multiplicity-adjusted. Significance was set at α = 0.05, with two-sided t-tests and coefficient tests.

## 3 Results

### 3.1 Participant characteristics

Characteristics of the analytic sample (*n* = 512) are presented in Table 1. For participants with numeric age entries (*n* = 511), mean age was 20.28 years (SD 2.61; range 18–39). The mean PPS total score was 40.80 (SD 7.32), with mean scores of 15.04 (SD 2.86) for the Work and Employment subscale and 25.77 (SD 4.84) for the Socio-Economic and Political subscale.

**Table 1:** Sociodemographic characteristics and perceived causes of poverty (*n* = 512).

Variable	Category	*n*	%
Age (years)	*n* = 511; mean ± SD 20.28 ± 2.61; range 18–39		
Gender	Woman	362	70.7
	Man	150	29.3
Employment status	Yes	42	8.2
	No	470	91.8
Year of study	Year 1	332	64.8
	Year 2	180	35.2
Family income level	Income < expenses	118	23.0
	Income = expenses	338	66.0
	Income > expenses	56	10.9
Father’s education level	No formal schooling (illiterate)	14	2.7
	Primary school	143	27.9
	Middle school	104	20.3
	High school	150	29.3
	Bachelor’s degree	82	16.0
	Postgraduate degree	19	3.7
Mother’s education level	No formal schooling (illiterate)	86	16.8
	Primary school	218	42.6
	Middle school	74	14.5
	High school	103	20.1
	Bachelor’s degree	24	4.7
	Postgraduate degree	7	1.4
Mother’s employment status	Yes	61	11.9
	No	451	88.1
Father’s employment status	Yes	405	79.1
	No	107	20.9
Which factors cause poverty?	Personal problems/illness	47	9.2
	Injustice	220	43.0
	Lack of opportunity	141	27.5
	Fate	49	9.6
	Sometimes people are to blame	55	10.7

The cause-of-poverty item was single-response; category frequencies sum to 512.

A free-text item asked respondents to name their clinical placement or work setting. A setting was named in 331 questionnaires (64.6%); 34 (6.6%) contained a response that did not identify a setting (e.g., “none,” “not working,” “student” or a single character), and the item was left blank in 147 (28.7%). The item did not quantify clinical exposure and was reported descriptively rather than included as a regression covariate.

### 3.2 Perceived causes of poverty

In the single-response item, “injustice” was the most frequently selected option (n = 220, 43.0%), followed by “lack of opportunity” (*n* = 141, 27.5%), “sometimes people are to blame” (*n* = 55, 10.7%), “fate” (*n* = 49, 9.6%) and “personal problems/illness” (*n* = 47, 9.2%). Each of the 512 analysed records contained one response.

### 3.3 Comparisons by participant characteristics

Group comparisons for the PPS total score are presented in Table 2 and subscale comparisons in Supplementary Table S3. Women had higher mean total scores than men (41.22, SD 7.10 vs. 39.81, SD 7.77; t (510) = 1.99, *p* = 0.048; Hedges’ g = 0.19, 95% CI 0.001 to 0.38).

**Table 2:** Total Perception of Poverty Scale score by participant characteristics (*n* = 512).

Variable/group	*n*	Total score (M ± SD)	Test	*p*	Effect size [95% or 90% CI]
Gender: woman vs. man	362/150	41.22 ± 7.10/39.81 ± 7.77	t (510) = 1.99	0.048	g = 0.19 [0.001, 0.38]
Employment: yes vs. no	42/470	39.38 ± 7.62/40.93 ± 7.29	t (510) = −1.31	0.189	g = −0.21 [−0.53, 0.11]
Mother employed: yes vs. no	61/451	40.95 ± 7.82/40.78 ± 7.26	t (510) = 0.17	0.867	g = 0.02 [−0.25, 0.29]
Father employed: yes vs. no	405/107	40.83 ± 7.21/40.70 ± 7.77	t (510) = 0.16	0.872	g = 0.02 [−0.20, 0.23]
Year of study: 1 vs. 2	332/180	40.52 ± 7.27/41.33 ± 7.41	t (510) = −1.21	0.228	g = −0.11 [−0.29, 0.07]
Family income level			Welch *F* (2, 131.4) = 0.13	0.874	η^2^p = 0.001 [0.000, 0.004]
Income < expenses	118	41.06 ± 8.24			
Income = expenses	338	40.78 ± 7.03			
Income > expenses	56	40.43 ± 7.14			
Father’s education level			*F* (5, 506) = 0.98	0.426	η^2^p = 0.010 [0.000, 0.018]
No formal schooling	14	40.00 ± 8.52			
Primary school	143	41.78 ± 7.18			
Middle school	104	40.81 ± 7.06			
High school	150	40.47 ± 7.04			
Bachelor’s degree	82	40.30 ± 7.57			
Postgraduate degree	19	38.74 ± 9.79			
Mother’s education level			F (5, 506) = 3.27	0.006	η^2^p = 0.031 [0.005, 0.051]
No formal schooling	86	41.92 ± 7.43			
Primary school	218	41.60 ± 7.05			
Middle school	74	40.88 ± 7.62			
High school	103	38.89 ± 7.15			
Bachelor’s degree	24	38.83 ± 7.45			
Postgraduate degree	7	36.29 ± 7.27			
Tukey HSD: high school vs. primary school				0.023	g = −0.38 [−0.62, −0.14]
Which factors cause poverty?			*F* (4, 507) = 5.24	< 0.001	η^2^p = 0.040 [0.012, 0.065]
Personal problems/illness	47	40.38 ± 6.54			
Injustice	220	42.45 ± 7.15			
Lack of opportunity	141	39.43 ± 7.54			
Fate	49	39.59 ± 7.75			
Sometimes people are to blame	55	39.18 ± 6.55			
Tukey HSD: injustice vs. lack of opportunity				0.001	g = 0.41 [0.20, 0.63]
Tukey HSD: injustice vs. “sometimes people are to blame”				0.023	g = 0.46 [0.16, 0.76]

The total score is the primary outcome; subscale comparisons are reported in Supplementary Table S3. Hedges’ g is signed in the direction first-named group minus second-named group and is reported with its 95% CI; partial eta squared (η^2^p) is reported with its 90% CI. Welch’s ANOVA was used where Levene’s test was significant (family income, *p* = 0.017). Pairwise contrasts are Tukey HSD comparisons of the two named groups; all other pairwise contrasts were non-significant (*p* ≥ 0.0502 for maternal education; *p* ≥ 0.090 for cause of poverty).

Scores differed across maternal-education groups (*F* (5, 506) = 3.27, *p* = 0.006; partial η^2^ = 0.031, 90% CI 0.005 to 0.051). Tukey’s HSD identified one significant pairwise difference: students whose mothers had completed high school scored lower than those whose mothers had completed primary school (38.89, SD 7.15 vs. 41.60, SD 7.05; mean difference −2.71, Tukey-adjusted *p* = 0.023; g = −0.38, 95% CI − 0.62 to −0.14). The high-school versus no-formal-schooling comparison was not statistically significant (Tukey-adjusted *p* = 0.0502), and no comparison involving the postgraduate group (*n* = 7) was significant (all Tukey-adjusted *p* ≥ 0.36). A Welch sensitivity analysis yielded a similar result (*F* (5, 51.1) = 3.13, *p* = 0.015). PPS scores were negatively correlated with ordinal maternal education (Pearson r = −0.168, *p* < 0.001; Spearman ρ = −0.143, *p* = 0.001).

No statistical evidence of differences in PPS scores was found across paternal-education groups (*F* (5, 506) = 0.98, *p* = 0.426; partial η^2^ = 0.010) or family income groups (Welch *F*(2, 131.4) = 0.13, *p* = 0.874). The Pearson correlation with ordinal paternal education was −0.080 (*p* = 0.071). No statistically significant differences were observed by year of study (*p* = 0.228), students’ employment status (*p* = 0.189), or maternal or paternal employment status (*p* = 0.867 and 0.872, respectively).

Scores also differed by the selected cause of poverty (*F* (4, 507) = 5.24, *p* < 0.001; partial η^2^ = 0.040, 90% CI 0.012 to 0.065). Tukey’s HSD identified two significant pairwise differences. Students selecting “injustice” (42.45, SD 7.15) scored higher than those selecting “lack of opportunity” (39.43, SD 7.54; mean difference 3.02, Tukey-adjusted *p* = 0.001; g = 0.41, 95% CI 0.20 to 0.63) and those selecting “sometimes people are to blame” (39.18, SD 6.55; mean difference 3.27, Tukey-adjusted *p* = 0.023; g = 0.46, 95% CI 0.16 to 0.76). All remaining Tukey comparisons were non-significant (adjusted *p* ≥ 0.090).

### 3.4 Factors associated with poverty perception

Hierarchical regression results are presented in Table 3. R^2^ increased from 0.011 for gender and year of study to 0.038 after adding income adequacy and maternal and paternal education (ΔR^2^ = 0.027, ΔF (3, 506) = 4.68, *p* = 0.003), and to 0.075 after adding the injustice-versus-other attribution contrast (ΔR^2^ = 0.038, ΔF(1, 505) = 20.67, *p* < 0.001). The final model accounted for 7.5% of the variance in PPS scores (adjusted R^2^ = 0.064; *F* (6, 505) = 6.86, *p* < 0.001).

**Table 3:** Factors associated with Perception of Poverty Scale scores: hierarchical multiple linear regression (*n* = 512).

Predictor	B	SE	β	t	*p*	95% CI
*Model 1 — R^2^ = 0.011, adjusted R^2^ = 0.007, F (2, 509) = 2.78, p = 0.063*						
Gender (female; ref. male)	1.44	0.74	0.089	1.94	0.052	−0.01 to 2.89
Year of study	0.86	0.68	0.056	1.27	0.206	−0.47 to 2.19
*Model 2 — R^2^ = 0.038, adjusted R^2^ = 0.028, F (5, 506) = 3.95, p = 0.002; ΔR^2^ = 0.027, ΔF (3, 506) = 4.68, p = 0.003*						
Gender (female)	1.41	0.73	0.088	1.94	0.053	−0.02 to 2.84
Year of study	0.59	0.68	0.039	0.87	0.382	−0.74 to 1.92
Income below expenses (ref. equal or above)	0.09	0.84	0.005	0.10	0.919	−1.56 to 1.73
Mother’s education (1–6)	−1.09	0.32	−0.178	−3.41	< 0.001	−1.72 to −0.46
Father’s education (1–6)	0.16	0.34	0.028	0.49	0.624	−0.50 to 0.82
*Model 3 — R^2^ = 0.075, adjusted R^2^ = 0.064, F (6, 505) = 6.86, p < 0.001, f^2^ = 0.082; ΔR^2^ = 0.038, ΔF (1, 505) = 20.67, p < 0.001*						
Gender (female)	1.29	0.71	0.080	1.80	0.072	−0.11 to 2.69
Year of study	0.49	0.67	0.032	0.74	0.459	−0.82 to 1.81
Income below expenses	0.11	0.84	0.006	0.13	0.899	−1.54 to 1.75
Mother’s education (1–6)	−1.15	0.31	−0.188	−3.67	< 0.001	−1.77 to −0.53
Father’s education (1–6)	0.23	0.33	0.039	0.71	0.476	−0.41 to 0.88
Attribution to injustice (ref. all other responses)	2.88	0.64	0.195	4.49	< 0.001	1.62 to 4.15

Dependent variable: total Perception of Poverty Scale score. Model 3 constant B = 40.12 (SE = 1.50). Standard errors are HC3 heteroscedasticity-consistent; t statistics, p-values and 95% confidence intervals for the coefficients use the t distribution with residual degrees of freedom. Model-level F and ΔF tests are ordinary least-squares tests. Confidence intervals are for unstandardised coefficients; standardised coefficients (β) are point estimates. VIF 1.01–1.50; Durbin–Watson = 2.05; maximum Cook’s distance = 0.040. Year of study was entered at its reported value (1 or 2); parental education was entered as an ordinal variable (1 = no formal schooling to 6 = postgraduate degree). Sensitivity analyses are reported in Supplementary Tables S1, S2.

In the final model, each one-category increase in maternal education, coded 1–6, was associated with a 1.15-point lower PPS score (B = −1.15, 95% CI − 1.77 to −0.53; β = −0.188, *p* < 0.001). Students selecting “injustice” scored 2.88 points higher than the combined group selecting other explanations (B = 2.88, 95% CI 1.62 to 4.15; β = 0.195, *p* < 0.001). The adjusted female–male difference was 1.29 points (95% CI − 0.11 to 2.69, *p* = 0.072), compared with 1.41 points before adjustment. The confidence intervals for year of study (B = 0.49, 95% CI − 0.82 to 1.81), income below versus equal to/above expenses (B = 0.11, 95% CI − 1.54 to 1.75) and paternal education (B = 0.23, 95% CI − 0.41 to 0.88) included zero.

Sensitivity analyses using categorical coding indicated associations with PPS scores for maternal education (*F* (5, 498) = 2.89, *p* = 0.014) and poverty attribution (*F*(4, 498) = 5.29, *p* < 0.001; Supplementary Table S2). An HC3 Wald test did not detect departure from the ordinal maternal-education coding (*F* (4, 498) = 0.37, *p* = 0.83). Category-specific estimates and covariate-adjusted contrasts are reported in Supplementary Table S2; the contrasts were not adjusted for multiple comparisons. The postgraduate maternal-education estimate was based on seven students and had a wide confidence interval (B = −6.46, 95% CI − 12.67 to −0.26).

With income entered as three categories (reference: income equal to expenses), R^2^ remained 0.075 to three decimal places; the coefficients for income below and above expenses had *p*-values of 0.900 and 0.996, respectively (Supplementary Table S1).

In a separate comparison excluding the poverty-attribution variable, adding maternal education to a model containing gender, year of study, income adequacy and paternal education increased R^2^ from 0.016 to 0.038 (ΔR^2^ = 0.021, ΔF (1, 506) = 11.17, *p* = 0.001). The paternal-education coefficient changed from −0.43 to 0.16; maternal and paternal education were correlated (r = 0.57).

## 4 Discussion

This study identified two associations with PPS scores among first- and second-year nursing students: an inverse association with maternal education and higher scores among students selecting “injustice” as the cause of poverty. No evidence of an association with self-reported household income adequacy was found. The exploratory model explained 7.5% of the variance, indicating that the measured characteristics captured only a limited part of the variation in PPS scores.

### 4.1 Perceptions of the causes of poverty

“Injustice” and “lack of opportunity” were the most frequently selected explanations, at 43.0 and 27.5%, respectively. This pattern suggests an emphasis on social constraints, but it does not establish a validated structural-attribution profile. The single-response format also cannot show whether students endorsed several explanations simultaneously. In an earlier Turkish study, nursing students endorsed fatalistic explanations more strongly than social-work students (7). That within-study disciplinary comparison is not directly equivalent to the distribution observed here. Differences in instruments, response formats and sampled cohorts limit conclusions about changes over time or differences between institutions. More recently, Rutar et al. (17) reported that injustice and lack of opportunities were each selected by 28.6% of the 56 nursing students in a Slovenian survey. Differences between this distribution and the present findings should be considered alongside differences in samples and instruments.

International evidence also illustrates the importance of distinguishing the constructs being compared. Scheffer et al. (8) found more favourable attitudes towards poverty and social justice among US than UK nursing students, but measured attitudes rather than the PPS-defined beliefs examined here. In the present study, students selecting “injustice” had higher PPS scores than those selecting “lack of opportunity”. Shek (18) identified exploitation and lack of opportunity as separate dimensions in a multi-item measure of poverty explanations. That finding does not validate the present response options or establish that “injustice” is equivalent to exploitation. The observed difference therefore warrants investigation with multi-item measures rather than interpretation as evidence of two distinct constructs.

### 4.2 Characteristics associated with perception

The income finding applies specifically to perceived household income adequacy, not to socioeconomic position as a whole. In Swiss panel data, Marquis et al. (15) found that relationships with poverty attributions varied across indicators of social stratification, while changes in stratification did not explain short-term within-person changes in attributions. Their measures and population differ from those of the present study. Our single subjective income item cannot establish whether objective income, material deprivation or other aspects of family circumstances are associated with PPS scores.

The inverse association with maternal education should not be interpreted as a protective effect or as evidence of better understanding of poverty. Lower PPS scores indicate less agreement with the scale’s statements (13), not necessarily poorer knowledge or greater stigma. The only significant Tukey comparison was between the high-school and primary-school groups, and estimates for the seven students whose mothers held postgraduate degrees were imprecise. The categorical analysis did not detect departure from the ordinal coding, but did not establish a linear relationship or an educational threshold. Özpinar and Akdede (9) associated adults’ own education with structural rather than fatalistic explanations in three Turkish cities; this does not directly explain the parental-education association observed here. Maternal and paternal education were correlated. Unmeasured family beliefs, contact with poverty or differential participation are possible explanations, but were not tested in this study.

The association between selecting “injustice” and PPS scores may partly reflect overlapping content: both the attribution item and the Socio-Economic and Political subscale concern explanations of poverty. Shared response tendencies may also contribute. Because the measures were collected concurrently, the findings cannot establish direction or demonstrate that changing an attribution would change broader perceptions. The female–male difference remained small after adjustment, with a confidence interval including zero; gender-specific pathways were not assessed. Similarly, the comparison of two year-of-study cohorts cannot describe change across the nursing programme.

### 4.3 Implications for nursing education

The findings identify students’ interpretations of injustice and lack of opportunity as topics for critical reflection and educational research. They do not establish that these topics should take priority over other curricular content or support for students’ own financial difficulties. Nor do they justify selecting students for interventions on the basis of maternal education. Poverty-related teaching should be evaluated as curricular provision rather than prescribed to demographic groups on the basis of these associations.

Existing educational research offers options for evaluation, but not uniform evidence of benefit. Holland et al. (11) described reflections on empathy and social justice in nursing students’ accounts following a poverty simulation. In a quasi-experimental study, Kruse and El-Khoury (12) found outcome-dependent changes, with social-justice attitudes improving only in the non-simulation comparison group. Sanko et al. (10) did not detect an overall difference in change between two simulation formats, despite differences on selected subscales. Doyle (19) reported higher social-justice attitude scores after a clinical rotation involving adults with low incomes. These findings concern different outcomes and designs; they do not establish sustained improvements in clinical practice.

Accordingly, controlled evaluations should assess knowledge, stigma and observable practice alongside perception scores, and examine whether changes persist. The PPS was developed to measure university students’ perceptions of poverty (13); increasing its total score should not itself be treated as evidence of successful teaching. Following students through clinical placements would also help distinguish within-student change from differences between cohorts.

### 4.4 Limitations

The survey did not restrict repeated submissions; duplicate responses therefore cannot be ruled out. The cross-sectional design does not support causal inference, and the model accounted for only a small share of between-student variation. Recruitment at one university through a voluntary-response online survey limits generalisability and permits self-selection bias. Before age-based exclusions, questionnaire returns corresponded to 87.8% of first-year enrolment and 62.4% of second-year enrolment. Senior students were not included, and the free-text placement or work-setting item did not quantify the extent of clinical exposure.

Measurement limitations include the single subjective income-adequacy item, the single-response attribution item and the equal-spacing assumption for ordinal parental education. The small upper-education groups limited the precision of category-specific estimates. The study assessed PPS-defined perceptions, not perceptions of poverty-related health inequalities. The strong correlations between subscale scores (r = 0.80) and latent factors (0.96), together with content shared by the attribution item and PPS, limit interpretation of subscale differences as evidence about distinct constructs. The reported internal consistency and factor analysis do not establish that PPS scores measure knowledge, stigma or clinical competence. All responses were self-reported and may be affected by social desirability.

Finally, the regression model was specified after initial analyses had been examined, and all analyses were exploratory. Multiplicity adjustment was limited to Tukey comparisons within each grouping. Findings from other comparisons and sensitivity analyses should therefore be treated as hypothesis-generating rather than confirmatory.

## 5 Conclusion

Among early-year nursing students at one Turkish university, “injustice” was the most frequently selected explanation of poverty. Higher maternal education was associated with lower PPS scores, whereas selecting “injustice” was associated with higher scores; no evidence of an association with household income adequacy was found. These exploratory findings do not demonstrate the effectiveness of nursing education. Further research should follow students across study years and clinical placements, use multi-item attribution measures, and evaluate poverty-related teaching through knowledge, stigma and practice outcomes rather than perception scores alone.

## Data Availability

The original contributions presented in the study are included in the article/Supplementary material, further inquiries can be directed to the corresponding author.

## References

[1] Suppa N Kanagaratnam U. The global multidimensional poverty index: harmonised level estimates and their changes over time. Sci Data. (2025) 12:153. doi: 10.1038/s41597-024-04269-x, 39865076 PMC11770090

[2] World Health Organization World Report on social Determinants of Health Equity. Geneva: World HealthOrganization (2025)

[3] Wang Q Pan X Li C Zheng F Zhang Z Dong X . Social determinants of health and life expectancy in US adults. J Transl Med. (2025) 24:40. doi: 10.1186/s12967-025-07488-x, 41340072 PMC12781748

[4] Wakefield MK Williams DR Le Menestrel S Lalitha J. The Future of Nursing 2020–2030: Charting a path to Achieve Health Equity. *Natl Acad Sci*. (2021) 10:25982. doi: 10.17226/25982 34524769

[5] Schneiderman JU Olshansky EF. Nurses’ perceptions: addressing social determinants of health to improve patient outcomes. Nurs Forum. (2021) 56:313–321. doi: 10.1111/nuf.12549 33484172

[6] Reutter LI Sword W Meagher-Stewart D Rideout E. Nursing students’ beliefs about poverty and health. J Adv Nurs. (2004) 48:299–309. doi: 10.1111/j.1365-2648.2004.03199.x, 15488044

[7] Demirtas B Polat G Özalp Ateş FS Ka ‘opua LS. Poverty and health inequalities: perceptions of social work students and nursing students. Int Nurs Rev. (2022) 69:96–105. doi: 10.1111/inr.12714, 34547107

[8] Scheffer MM Lasater K Atherton IM Kyle RG. Student nurses' attitudes to social justice and poverty: an international comparison. Nurse Educ Today. (2019) 80:59–66. doi: 10.1016/j.nedt.2019.06.007, 31271940

[9] Özpinar Ş Akdede SH. Determinants of the attribution of poverty in Turkey: an empirical analysis: S. Özpinar, SH Akdede. Soc Indic Res. (2022) 164:949–67. doi: 10.1007/s11205-022-02988-5 PMC9358094 35968219

[10] Sanko JS Matsuda Y Salani D Tran L Reaves R Gerber K. A comparison of learning outcomes from two poverty simulation experiences. Public Health Nurs. (2021) 38:427–38. doi: 10.1111/phn.12853, 33410560

[11] Holland T Walter L Langevin K Bourgoin A McKelvey M Kirk C . Nursing students’ experience of a poverty simulation and its impact on empathy and social justice awareness: a descriptive qualitative study. Creat Nurs. (2024) 30:65–73. doi: 10.1177/10784535241229143, 38304931

[12] Kruse JA El-Khoury H. A poverty simulation to enhance nursing student knowledge, skills, and attitudes toward people living in poverty. Nurs Forum. (2022) 57:851–859. doi: 10.1111/nuf.12739 35641886

[13] Uru MK Dirimeşe E. Üniversite Öğrencileri Yoksulluk Algısı Ölçeği Geliştirme Çalışması. MANAS Sosyal Arastirmalar Derg. (2024) 13:613–28. doi: 10.33206/mjss.1270295

[14] Smithson M. Correct confidence intervals for various regression effect sizes and parameters: the importance of noncentral distributions in computing intervals. Educ Psychol Meas. (2001) 61:605–32. doi: 10.1177/00131640121971392

[15] Marquis L Kuhn U Tillmann R. Causal attributions of poverty: a social stratification analysis. Front Sociol. (2025) 10:1591235. doi: 10.3389/fsoc.2025.1591235, 40556861 PMC12185453

[16] MacKinnon JG White H. Some heteroskedasticity-consistent covariance matrix estimators with improved finite sample properties. J Econom. (1985) 29:305–25. doi: 10.1016/0304-4076(85)90158-7

[17] Rutar P Prosen M Ličen S. Health science students' perceptions of poverty and health inequality. Kontakt. (2024) 26:354–60. doi: 10.32725/kont.2024.056

[18] Shek DTL. Chinese people’s explanations of poverty: the perceived causes of poverty scale. Res Soc Work Pract. (2003) 13:622–40. doi: 10.1177/1049731503253376, 12564829

[19] Doyle BA. Social justice attitudes of undergraduate nursing students: the impact of praxis. Public Health Nurs. (2023) 40:734–9. doi: 10.1111/phn.13219, 37312592

