# Supplementary material for: Perceptions of poverty and associated factors among first- and second-year nursing students: a cross-sectional study
Source: Front Public Health. 2026 Sep 23;14:1941236. doi: 10.3389/fpubh.2026.1941236 (PMC13642323; doi:10.3389/fpubh.2026.1941236)
Supplement: Supplementary file 1 [file Table_1.DOCX]

**Supplementary Material**

This file contains Supplementary Tables S1–S3. S1 and S2 are sensitivity analyses of the hierarchical regression reported in Table 3 of the main text (three-category coding of family income; categorical coding of maternal education and of the causal-attribution item). S3 reports the group comparisons of Table 2 for the two subscale scores, which are secondary outcomes. All analyses use the analytic sample of 512 participants described in Section 2.1 and follow the analytic conventions stated in Section 2.4 (HC3 standard errors with t-distribution inference for regression coefficients; Welch’s tests where Levene’s test was significant; Hedges’ g with 95% CI and partial eta squared with 90% CI). Abbreviations: B, unstandardised coefficient; SE, standard error; β, standardised coefficient; CI, confidence interval; PPS, Perception of Poverty Scale for University Students.

**SUPPLEMENTARY TABLE S1 Sensitivity analysis: three-category family income (n = 512)**

| **Predictor** | **B** | **SE** | **β** | **t** | **p** | **95% CI** |
| --- | --- | --- | --- | --- | --- | --- |
| Gender (female; ref. male) | 1.29 | 0.72 | .080 | 1.80 | .072 | −0.12 to 2.69 |
| Year of study | 0.49 | 0.67 | .032 | 0.74 | .461 | −0.82 to 1.81 |
| Income below expenses (ref. income = expenses) | 0.11 | 0.85 | .006 | 0.13 | .900 | −1.56 to 1.77 |
| Income above expenses (ref. income = expenses) | 0.00 | 1.04 | .000 | 0.00 | .996 | −2.04 to 2.05 |
| Mother’s education (1–6) | −1.15 | 0.31 | −.188 | −3.66 | < .001 | −1.77 to −0.53 |
| Father’s education (1–6) | 0.23 | 0.33 | .039 | 0.71 | .477 | −0.41 to 0.88 |
| Attribution to injustice (ref. all other responses) | 2.88 | 0.65 | .195 | 4.47 | < .001 | 1.62 to 4.15 |

Note. R² = .075, adjusted R² = .063, F(7, 504) = 5.87, p < .001. Constant B = 40.12 (SE = 1.51). HC3 standard errors; t-distribution p-values and confidence intervals. All predictors in the model are shown.

**SUPPLEMENTARY TABLE S2 Sensitivity analysis: categorical coding of maternal education and causal attribution (n = 512)**

| **Predictor** | **B** | **SE** | **β** | **t** | **p** | **95% CI** |
| --- | --- | --- | --- | --- | --- | --- |
| Gender (female; ref. male) | 1.33 | 0.73 | .082 | 1.81 | .071 | −0.11 to 2.77 |
| Year of study | 0.50 | 0.68 | .033 | 0.74 | .463 | −0.84 to 1.84 |
| Income below expenses (ref. equal or above) | 0.14 | 0.87 | .008 | 0.16 | .870 | −1.57 to 1.85 |
| Father’s education (1–6) | 0.21 | 0.33 | .035 | 0.64 | .525 | −0.44 to 0.86 |
| *Maternal education (ref. no formal schooling)* | | | | | | |
| Primary school | −0.76 | 1.00 | −.052 | −0.77 | .444 | −2.72 to 1.20 |
| Middle school | −1.34 | 1.27 | −.064 | −1.06 | .292 | −3.82 to 1.15 |
| High school | −3.58 | 1.19 | −.196 | −3.01 | .003 | −5.92 to −1.25 |
| Bachelor’s degree | −3.55 | 1.86 | −.103 | −1.91 | .056 | −7.20 to 0.09 |
| Postgraduate degree | −6.46 | 3.16 | −.103 | −2.05 | .041 | −12.67 to −0.26 |
| *Causal attribution (ref. personal problems / illness)* | | | | | | |
| Injustice | 2.28 | 1.13 | .155 | 2.02 | .044 | 0.06 to 4.51 |
| Lack of opportunity | −0.95 | 1.19 | −.058 | −0.81 | .421 | −3.28 to 1.37 |
| Fate | −0.48 | 1.49 | −.019 | −0.32 | .746 | −3.41 to 2.44 |
| Sometimes people are to blame | −0.32 | 1.39 | −.014 | −0.23 | .819 | −3.05 to 2.42 |

Note. R² = .080, adjusted R² = .056, F(13, 498) = 3.31, p < .001. Constant B = 39.30 (SE = 1.82). HC3 standard errors; t-distribution p-values and confidence intervals. Maternal education block: ΔR² = .027, F(5, 498) = 2.89, p = .014; causal attribution block: ΔR² = .039, F(4, 498) = 5.29, p < .001. Test of departure from the linear (ordinal) coding of maternal education (four restrictions, HC3 covariance): Wald F(4, 498) = 0.37, p = .832. Adjusted contrasts between attribution categories estimated from this model (HC3): injustice vs lack of opportunity B = 3.24, 95% CI 1.69 to 4.79, p < .001; injustice vs “sometimes people are to blame” B = 2.60, 95% CI 0.53 to 4.68, p = .014; injustice vs fate B = 2.77, 95% CI 0.49 to 5.05, p = .018; lack of opportunity vs “sometimes people are to blame” B = −0.63, 95% CI −2.86 to 1.59, p = .575. Maternal education, high school vs primary school: B = −2.82, 95% CI −4.59 to −1.05, p = .002. The reference category for attribution contains 47 participants and the bachelor’s and postgraduate maternal-education groups contain 24 and 7, which limits the precision of the corresponding estimates.

**SUPPLEMENTARY TABLE S3 Subscale scores by participant characteristics (n = 512)**

| **Variable / group (n)** | **Work and Employment: M ± SD; test; p; effect size [CI]** | **Socio-Economic and Political: M ± SD; test; p; effect size [CI]** |
| --- | --- | --- |
| Gender: woman (362) vs man (150) | 15.10 ± 2.83 vs 14.87 ± 2.93; t(510) = 0.86; p = .391; g = 0.08 [−0.11, 0.27] | 26.11 ± 4.63 vs 24.94 ± 5.25; Welch t(249.4) = 2.37; p = .018; g = 0.24 [0.05, 0.43]; Levene p = .026 |
| Employment: yes (42) vs no (470) | 14.48 ± 3.02 vs 15.09 ± 2.84; t(510) = −1.32; p = .186; g = −0.21 [−0.53, 0.10] | 24.90 ± 4.90 vs 25.84 ± 4.84; t(510) = −1.21; p = .228; g = −0.19 [−0.51, 0.12] |
| Mother employed: yes (61) vs no (451) | 14.89 ± 2.94 vs 15.06 ± 2.85; t(510) = −0.44; p = .663; g = −0.06 [−0.33, 0.21] | 26.07 ± 5.33 vs 25.73 ± 4.78; t(510) = 0.51; p = .609; g = 0.07 [−0.20, 0.34] |
| Father employed: yes (405) vs no (107) | 15.05 ± 2.82 vs 14.97 ± 3.01; t(510) = 0.26; p = .797; g = 0.03 [−0.19, 0.24] | 25.78 ± 4.78 vs 25.73 ± 5.08; t(510) = 0.09; p = .926; g = 0.01 [−0.20, 0.22] |
| Year of study: 1 (332) vs 2 (180) | 14.99 ± 2.87 vs 15.11 ± 2.85; t(510) = −0.44; p = .658; g = −0.04 [−0.22, 0.14] | 25.52 ± 4.78 vs 26.22 ± 4.93; t(510) = −1.57; p = .118; g = −0.14 [−0.33, 0.04] |
| Family income: < (118) / = (338) / > expenses (56) | 15.31 ± 3.01 / 14.97 ± 2.80 / 14.84 ± 2.90; F(2, 509) = 0.78; p = .460; η²p = .003 [.000, .013] | 25.75 ± 5.52 / 25.80 ± 4.61 / 25.59 ± 4.80; Welch F(2, 130.2) = 0.05; p = .951; η²p < .001 (90% CI at the boundary; see note); Levene p = .013 |
| Father’s education: no formal schooling (14) / primary (143) / middle (104) / high school (150) / bachelor’s (82) / postgraduate (19) | 14.79 ± 3.56 / 15.32 ± 2.69 / 15.14 ± 2.71 / 14.99 ± 2.77 / 14.77 ± 3.16 / 14.00 ± 3.61; F(5, 506) = 0.99; p = .424; η²p = .010 [.000, .018] | 25.21 ± 5.19 / 26.46 ± 4.76 / 25.66 ± 4.80 / 25.49 ± 4.72 / 25.54 ± 4.81 / 24.74 ± 6.36; F(5, 506) = 0.94; p = .452; η²p = .009 [.000, .018] |
| Mother’s education: no formal schooling (86) / primary (218) / middle (74) / high school (103) / bachelor’s (24) / postgraduate (7) | 15.47 ± 2.77 / 15.32 ± 2.78 / 15.30 ± 2.96 / 14.21 ± 2.76 / 14.21 ± 3.19 / 13.14 ± 2.85; F(5, 506) = 3.75; p = .002; η²p = .036 [.008, .057]; Welch F(5, 51.0) = 3.55, p = .008; Tukey HSD: no formal schooling vs high school p = .030 (g = 0.45, 95% CI 0.16 to 0.74); primary vs high school p = .015 (g = 0.40, 95% CI 0.16 to 0.63) | 26.45 ± 4.93 / 26.28 ± 4.73 / 25.58 ± 4.99 / 24.68 ± 4.74 / 24.62 ± 4.75 / 23.14 ± 4.53; F(5, 506) = 2.62; p = .024; η²p = .025 [.002, .043]; Welch F(5, 51.3) = 2.57, p = .038; Tukey HSD: primary vs high school p = .060 (g = 0.34, 95% CI 0.10 to 0.57) |
| Cause of poverty: personal problems / illness (47) / injustice (220) / lack of opportunity (141) / fate (49) / sometimes people are to blame (55) | 14.87 ± 2.73 / 15.50 ± 2.75 / 14.61 ± 3.04 / 14.67 ± 2.80 / 14.73 ± 2.79; F(4, 507) = 2.66; p = .032; η²p = .021 [.001, .038]; Tukey HSD: injustice vs lack of opportunity p = .031 (g = 0.31, 95% CI 0.10 to 0.52) | 25.51 ± 4.06 / 26.95 ± 4.78 / 24.82 ± 4.85 / 24.92 ± 5.26 / 24.45 ± 4.36; F(4, 507) = 6.31; p < .001; η²p = .047 [.017, .075]; Tukey HSD: injustice vs lack of opportunity p < .001 (g = 0.44, 95% CI 0.23 to 0.66); injustice vs fate p = .054 (g = 0.42, 95% CI 0.10 to 0.73); injustice vs “sometimes people are to blame” p = .005 (g = 0.53, 95% CI 0.23 to 0.83) |

Note. Subscale analyses are secondary and exploratory; Tukey-adjusted p-values are reported for pairwise comparisons within each grouping, with no adjustment across separate analyses, and these results are hypothesis-generating. Welch’s test was used where Levene’s test was significant (gender, p = .026, and family income, p = .013, on the Socio-Economic and Political subscale). Hedges’ g is signed first-named minus second-named group with approximate, pointwise 95% CI calculated using the t-based method in pingouin; η²p is reported with 90% CI obtained by inversion of the noncentral F distribution from the conventional ANOVA, including when a Welch test is reported. These effect-size intervals are not adjusted for multiple comparisons. For the Socio-Economic and Political income comparison, the inversion returned both bounds at zero; this boundary result should not be interpreted as precise evidence of no association. The two subscales are highly correlated (r = .80) and are not interpreted as distinct constructs.
